# Patent foramen ovale with cryptogenic stroke: A case report

**DOI:** 10.3892/mi.2024.204

**Published:** 2024-11-13

**Authors:** Dhruvil Vinaybhai Patel, Chandan Alenahalli Narayana, Rachana Harish, Harsh Bhatia, Shubh Mehta, Om Prakash Bhatta, Manjeet Singh

**Affiliations:** 1 Depatrment of Neurology, GMERS Medical College Gandhinagar, Gandhinagar, Gujarat 382012, India; 2 Bangalore Medical College and Research Institute, Bengaluru, Karnataka 560002, India; 3 Surat Municipal Institute of Medical Education and Research, Surat, Gujarat 395010, India; 4 B.J. Medical College and Civil Hospital, Ahmedabad, Gujarat 380016, India; 5 Department of Anesthesiology, Tribhuvan University Teaching Hospital, Kathmandu, Nepal 44600, India; 6 Liaquat National Medical College, Karachi, Sindh 74800, Pakistan

**Keywords:** patent foramen ovale, cryptogenic stroke, ischemic stroke, percutaneous closure, antiplatelet therapy

## Abstract

Patent foramen ovale (PFO) is a common congenital heart defect that can contribute to cryptogenic stroke, particularly in younger patients lacking traditional risk factors. The present study describes the case of a 14-year-old male patient who experienced an acute ischemic stroke without identifiable atheroembolic risk factors. Comprehensive diagnostic imaging revealed a marked right-to-left shunt through a PFO, identified as the probable cause of the stroke. The patient was treated with dual antiplatelet therapy and subsequently underwent successful percutaneous PFO closure. At 6 months following the procedure, the patient exhibited notable neurological recovery. The present case report highlights the critical need for evaluating PFO as a potential etiology of cryptogenic stroke in younger populations, emphasizing the advantages of a multidisciplinary approach and timely intervention in preventing recurrent events.

## Introduction

Patent foramen ovale (PFO) is a prevalent defect in the interatrial septum, occurring in 15-35% of the adult population (1). While the majority of individuals with a PFO remain asymptomatic throughout their lives, this condition has increasingly been recognized as a potential contributor to cryptogenic stroke, particularly in children and young adults without classic risk factors for atheroembolic stroke (2). Cryptogenic stroke accounts for 25-40% of all ischemic stroke cases, posing diagnostic and therapeutic challenges due to the absence of a clearly identifiable cause (3). In young patients with cryptogenic stroke, the prevalence of PFO can be as high as 50% (4).

The role of PFO in the development of cryptogenic stroke may involve promoting thrombus formation or acting as a pathway for paradoxical embolism, where a thrombus bypasses lung filtration and moves from venous to arterial circulation through the foramen ovale, potentially leading to cerebral ischemia. However, establishing a direct causal link between PFO and stroke can be complex, and the available evidence is often speculative and less well documented, necessitating a comprehensive diagnostic workup (1,5). Additionally, treatment options for PFO remain controversial due to the lack of clear practice guidelines and the risks associated with both invasive and non-invasive procedures.

The present study describes the case of a 14-year-old male patient who suffered an acute ischemic stroke without conventional risk factors. Following a thorough diagnostic workup that identified a marked right-to-left shunt through the PFO, the patient received dual antiplatelet therapy and underwent successful percutaneous PFO closure. Notably, the patient achieved substantial recovery at the 6-month follow-up, underscoring the importance of a multidisciplinary approach in diagnosing and managing PFO-related cryptogenic stroke in young patients.

## Case report

### Patient history

A 14-year-old male presented to the Emergency Department of GMERS Medical College (Gandhinagar, India) with sudden onset of right-sided weakness, slurred speech and facial drooping while playing soccer. His medical history was unremarkable, with no prior neurological deficits or cardiovascular issues. As a healthy, active teenager, he had no history of smoking, drug use, or a notable family history of cardiovascular or neurological diseases. Upon admission, his vital signs were within normal limits, and there were no signs of trauma or infection.

### Clinical examination

A physical examination revealed that the patient was alert and oriented; however, he exhibited right-sided hemiparesis (strength of 2/5 in the right upper and lower limbs), dysarthria and a right facial droop, leading to a National Institutes of Health Stroke Scale (NIHSS) score of 6. An urgent non-contrast computed tomography (CT) scan of the head revealed no evidence of intracranial hemorrhage or mass lesion. Given the age of the patient and absence of traditional stroke risk factors, a cryptogenic stroke was suspected. He was admitted to the pediatric intensive care unit (PICU) of GMERS Medical College (Gandhinagar, India) for monitoring and further workup.

### Diagnostic workup

**Figure 1:**
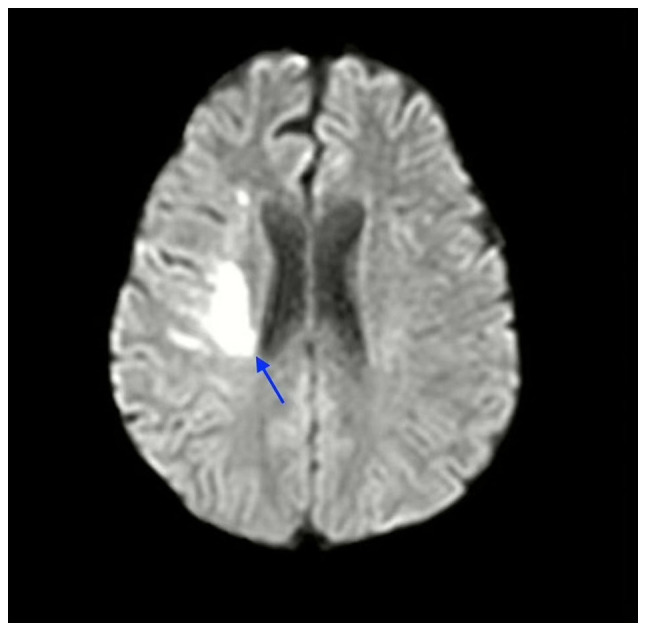
Magnetic resonance image of the brain of the patient, illustrating ischemic stroke (blue arrow).

Magnetic resonance imaging (MRI) of the brain with diffusion-weighted imaging (DWI) confirmed an acute ischemic infarct in the left middle cerebral artery (MCA) territory. A thorough diagnostic workup was initiated to identify potential underlying causes. Initial evaluation with a transthoracic echocardiogram (TTE) was normal; however, due to persistent stroke symptoms and the lack of identifiable risk factors, a transesophageal echocardiogram (TEE) was performed. This revealed a PFO with a marked right-to-left shunt, confirmed by a bubble study, while no other structural cardiac abnormalities were detected (Fig. 1 and Video S1).

A further evaluation included a hypercoagulability panel, which yielded negative results for any clotting disorders. Laboratory tests for autoimmune and infectious etiologies, including antiphospholipid antibodies and tests for vasculitis, also yielded negative results. The patient's lipid profile, fasting glucose (85 mg/dl), and hemoglobin A1c (5.4%) were all within normal limits, indicating no metabolic abnormalities contributing to his condition.

### Management

Throughout the diagnostic and treatment process, a multidisciplinary team consisting of specialists from cardiology, neurology and pediatrics played a pivotal role. Their collaborative discussions were instrumental in shaping the treatment management plan of the patient. i) Diagnostic collaboration: Neurologists were integral in interpreting the imaging studies and determining the acute ischemic nature of the stroke. Their insight prompted further cardiac evaluations, ultimately leading to the identification of the PFO through TEE. ii) Risk assessment and treatment planning: After confirming the diagnosis of a PFO and cryptogenic stroke, the team convened to discuss the treatment options. Neurologists advocated for immediate dual antiplatelet therapy with aspirin (81 mg daily) and clopidogrel (75 mg daily), while cardiologists emphasized the long-term benefits of PFO closure. This collaborative dialogue was critical in evaluating the risks and benefits of each option in the context of the age and clinical presentation of the patient. iii) Procedure decision: The consensus among the team was to proceed with percutaneous PFO closure to reduce the risk of recurrent stroke. This decision was made after weighing the potential complications of the procedure against the high risk of future stroke due to the identified shunt.

### Outcome

Following the multidisciplinary discussions, the patient underwent successful percutaneous closure of the PFO without complications. His neurological symptoms gradually improved with rehabilitation, and at the 6-month follow-up, he demonstrated a near-complete recovery, with only mild residual weakness in the right hand. Repeat imaging confirmed no new infarcts, and he continued on dual antiplatelet therapy with aspirin and clopidogrel for an additional 6 months postoperatively.

The case described herein highlights the critical need for a multidisciplinary approach in diagnosing and managing cryptogenic stroke in young patients. By integrating the expertise of various specialists, the team was able to provide a comprehensive assessment and implement timely interventions, ultimately improving the outcomes of the patient, and significantly reducing the risk of recurrent stroke.

## Discussion

The present case report describes the clinical features, diagnosis and treatment of a 14-year-old male patient who experienced a cryptogenic stroke, later attributed to a PFO. The clinical presentation of the patient was atypical, considering his age, the absence of traditional cardiovascular risk factors and the lack of a notable family history. Pediatric stroke cases are rare, with incidence rates ranging from 2.5 to 13 per 100,000 individuals per year (6). The case described herein offers a unique opportunity to explore the potential etiologies of pediatric stroke, including congenital heart defects, such as PFO, even in the absence of obvious predisposing factors.

A PFO is a congenital heart abnormality caused by the failure of antenatal interatrial communication to close, rendering it the most common cause of right-to-left shunting in adults. Autopsy studies report a prevalence of PFO between 14 and 35%, with a median of 26% and a weighted mean of 25% (7). This suggests that approximately two billion individuals worldwide have a persistent right-to-left communication (8). PFO is associated with various clinical syndromes, including cryptogenic stroke, decompression sickness, migraines and platypnea-orthodeoxia syndrome (9).

A cryptogenic stroke is defined as a stroke for which no clear cause can be identified following a thorough medical evaluation, accounting for 15-40% of all ischemic stroke cases (10). The association between PFO and cryptogenic stroke has been a matter of debate for several years, with some studies suggesting that nearly half of patients with cryptogenic strokes have a PFO (11). Estimates indicate that 40 to 50% of individuals experiencing a cryptogenic stroke may have this condition (11,12). While the literature indicates a significant link between PFO and stroke in older adults, particularly those >55 years of age, its association with pediatric populations has been less established (13-15). The present case report supports the need for a thorough cardiac evaluation in young patients with unexplained stroke (9,16).

The role of PFO in stroke remains unclear, with a continuing debate over whether it is a direct cause, a contributing risk factor, or merely an incidental finding. The previous meta-analysis by Alsheikh-Ali *et al* (17) estimated that approximately one-third of PFOs identified in patients with cryptogenic stroke were likely incidental and unrelated to the stroke. Despite this fact, the exact contribution of PFO to the pathogenesis of stroke, particularly in the pediatric populations, remains an active area of research, affecting clinical treatment approaches.

In the case that PFO is pathogenic, potential mechanisms for stroke include paradoxical embolism (where a venous thrombus crosses the PFO), in situ thrombus formation within the PFO, and atrial arrhythmias due to disrupted electrical signaling. The key risk factors for PFO-related stroke include young age, PFO size, the extent of the right-to-left shunt, PFO morphology and the presence of an atrial septal aneurysm. Additional factors, such as coagulation imbalances and other atrial anomalies (e.g., right atrial septal pouch, Eustachian valve, or Chiari network), can further increase the risk of embolic events, acting either independently or synergistically. Notably, the PFO in Cryptogenic Stroke Study (PICSS) found a higher prevalence of large PFOs in patients who suffered a cryptogenic stroke compared to those with identified stroke causes, suggesting that larger PFOs may be an independent risk factor for recurrent cerebrovascular events (5).

The diagnostic process in the case described herein highlights the challenges of identifying the underlying cause of stroke in young patients without clear risk factors, particularly regarding whether PFO is incidental or pathogenic, and the concerns related to deep vein thrombosis (DVT) and pulmonary thromboembolism (PTE) associated with PFO (18-20). Clergeau *et al* (18) reported that PFO independently increased the risk of silent brain ischemia (small, often undetected areas of brain damage) in patients who have experienced PTE.

However, the case in the present study is unique compared to others, such as the one presented by Park *et al* (19), which involved a massive PTE followed by DVT. In this instance, a CT scan of the head revealed no evidence of intracranial hemorrhage or mass lesion, while an MRI with DWI revealed an acute ischemic infarct in the left MCA territory. Further evaluation included a hypercoagulability panel, which yielded negative results for any clotting disorders. Additionally, tests for autoimmune and infectious etiologies, such as antiphospholipid antibodies, lupus anticoagulant and vasculitis, yielded negative results. The lipid profile, and fasting glucose and hemoglobin A1c levels of the patient were normal. This complexity suggests that while PFO may be a contributing factor, it does not conclusively establish a pathogenic role in this specific case. Further evidence is required to definitively prove PFO as the direct cause of the stroke.

TTE and TEE with saline contrast injection are commonly used to diagnose PFO. A PFO is confirmed if microbubbles appear in the left-sided cardiac chambers within three cardiac cycles after the right atrium reaches peak opacification (13). In the study by Pearson *et al* (20), transesophageal echocardiography detected a potential cardiac source of embolism in 57% of the participants, significantly higher than the 15% detection rate for TTE (P<0.0005). This was evident in the case in the present study, where initial normal findings on TTE could have led to an incomplete evaluation; however, subsequent TEE revealed the PFO. This underscores the importance of using advanced imaging techniques when initial assessments are inconclusive, particularly in cases where PFO is suspected (20,21).

The treatment approach for PFO in patients with cryptogenic stroke remains a matter of debate. Antithrombotic treatment options include antiplatelet drugs (e.g., aspirin and clopidogrel) and anticoagulants (warfarin, heparin and direct oral anticoagulants), with no notable difference in efficacy observed in the absence of atrial fibrillation (12). However, the recurrence rate for neurological events is higher with medication (5.0 events per 100 person-years) compared to PFO closure (0.8 events), indicating the superiority of the latter, particularly in patients <60 years of age (22,23).

Long-term follow-up data on PFO closure indicate significant benefits in preventing recurrent stroke and improving the quality of life of patients. The meta-analysis by Agarwal *et al* (22) demonstrated that patients who underwent transcatheter closure had a notably lower risk of recurrent neurological events compared to those receiving medical therapy alone, underscoring the efficacy of the procedure in the prevention of secondary stroke. Similarly, Lee *et al* (12) reported that patients with cryptogenic stroke and high-risk PFOs experienced reduced stroke recurrence rates post-closure, highlighting the importance of addressing this defect in affected individuals. Furthermore, Yaghi *et al* (3) emphasized improvements in the overall quality of life among patients following PFO closure, suggesting not only clinical benefits, but also enhancements in daily functioning and well-being. Additionally, the comprehensive analysis conducted by Sposato *et al* (8) in a multi-center registry confirmed the safety and sustained efficacy of PFO closure procedures over the long term, reinforcing the role of the procedure in the management of stroke. Collectively, these studies provide compelling evidence that PFO closure can lead to favorable long-term outcomes, advocating for its consideration in appropriate patient populations.

Neurologists often recommend closure for large shunts and atrial septal aneurysms, while cardiologists focus on thrombophilia cases. Post-closure, neurologists typically prefer long-term single antiplatelet therapy, whereas cardiologists may suggest dual therapy or no long-term treatment, highlighting the need for collaborative decision-making among specialties (24). Surgical intervention is rare, reserved for larger PFOs or when percutaneous closure fails (9,23). In the case in the present study, the patient began treatment with dual antiplatelet drugs, with aspirin and clopidogrel. Following multidisciplinary discussions, it was determined that PFO closure would aid in the prevention of future strokes.

In conclusion, the present case report demonstrates the crucial role of PFO as a potential cause of cryptogenic stroke in a young, otherwise healthy individual. Despite the absence of traditional stroke risk factors, the identification of a substantial right-to-left shunt through the PFO was crucial in diagnosing the underlying cause of the ischemic event. The successful percutaneous closure of the PFO, combined with dual antiplatelet therapy, resulted in a notable neurological recovery for the patient. This outcome emphasizes the importance of considering PFO in the differential diagnosis of cryptogenic stroke in younger patients and suggests that early intervention, including PFO closure, can be an effective strategy to prevent stroke recurrence and improve patient outcomes.

## Supplementary Material

2D echocardiographyillustrating blood flow through the patent foramen ovale.

Supplementary Data

## Data Availability

The datasets used and/or analyzed during the current study are available from the corresponding author on reasonable request.
