# Supplementary material for: Patent foramen ovale with cryptogenic stroke: A case report
Source: Med Int (Lond). 2024 Nov 13;5(1):5. doi: 10.3892/mi.2024.204 (PMC11609884; doi:10.3892/mi.2024.204)
Supplement: Supplementary Data [file Supplementary_Data2.pdf]

Video S1. 2D echocardiographyillustrating blood flow through the patent foramen ovale.
